# Reorganizing Pharmaceutical Care in Family Medicine Groups for Older Adults With or at Risk of Major Neurocognitive Disorders: Protocol for a Mixed Methods Study

**DOI:** 10.2196/42577

**Published:** 2022-11-17

**Authors:** Line Guénette, Edeltraut Kröger, Dylan Bonnan, Anne Maheu, Michèle Morin, Laurianne Bélanger, Isabelle Vedel, Machelle Wilchesky, Caroline Sirois, Etienne Durand, Yves Couturier, Nadia Sourial, Clémence Dallaire

**Affiliations:** 1 Faculty of Pharmacy Université Laval Québec, QC Canada; 2 Axe Santé des populations et pratiques optimales en santé Centre de recherche du CHU de Québec Université Laval Québec, QC Canada; 3 Centre d’excellence sur le vieillissement de Québec Centre intégré universitaire de santé et de services sociaux de la Capitale-Nationale Québec, QC Canada; 4 Centre intégré universitaire de santé et de services sociaux du Nord-de-l’Île-de-Montréal Montréal, QC Canada; 5 Faculté de médecine Université Laval Québec, QC Canada; 6 Centre intégré de santé et de services sociaux de Chaudière-Appalaches Sainte-Marie, QC Canada; 7 Department of Family Medicine McGill University Montréal, QC Canada; 8 Donald Berman Maimonides Centre for Research in Aging Montréal, QC Canada; 9 Division of Geriatric Medicine McGill University Montréal, QC Canada; 10 École de travail social Université de Sherbrooke Sherbrooke, QC Canada; 11 Department of Health Management, Evaluation and Policy School of Public Health University of Montreal Montréal, QC Canada; 12 Faculté des sciences infirmières Université Laval Québec, QC Canada

**Keywords:** primary care, older adults, neurocognitive disorders, pharmaceutical care, mixed method study

## Abstract

**Background:**

The latest global figures show that 55 million persons lived with major neurocognitive disorders (MNCDs) worldwide in 2021. In Quebec, Canada, most of these older adults are cared for by family physicians in interdisciplinary primary care clinics such as family medicine groups (FMG). When a person has a MNCD, taking potentially inappropriate medications or polypharmacy (5 different medications or more) increases their vulnerability to serious adverse events. With the recent arrival of pharmacists working in FMGs and their expanded scope of practice and autonomy, new possibilities for optimizing older adults’ pharmacotherapy are opening.

**Objective:**

This project aims to evaluate the impact of involving these pharmacists in the care trajectory of older adults living with MNCD, in an interdisciplinary collaboration with the FMG team, as well as home care nurses and physicians. Pharmacists will provide medication reviews, interventions, and recommendations to improve the pharmacotherapy and support offered to these patients and their caregivers.

**Methods:**

This 2-step mixed methods study will include a quasi-experimental controlled trial (step 1) and semistructured interviews (step 2). Older adults undergoing cognitive assessment, recently diagnosed with MNCD, or receiving care for this at home will be identified and recruited in FMGs in 2 Quebec regions. FMGs implementing the intervention will involve pharmacists in these patients’ care trajectory. Training and regular mentoring will be offered to these FMGs, especially to pharmacists. In control FMGs, no FMG pharmacist will be involved with these patients, and usual care will be provided.

**Results:**

Medication use (including appropriateness) and burden, satisfaction of care received, and quality of life will be assessed at study beginning and after 6 months of follow-up and compared between groups. At the end of the intervention study, we will conduct semistructured interviews with FMG care team members (pharmacists, nurses, and physicians) who have experienced the intervention. We will ask about the feasibility of integrating the intervention into practice and their satisfaction with and their perception of the intervention’s impacts for older adults and their families. We will assess the effect of improved pharmaceutical care for older adults with or at risk of MNCDs through the involvement of FMG pharmacists and a reorganization of pharmaceutical care.

**Conclusions:**

The inclusion of pharmacists in interdisciplinary care teams is recent and rising, strengthened by more substantial pharmacist practice roles. Results will inform the processes required to successfully involve pharmacists and implement developed tools and procedures transposable to other care settings to improve patient care.

**Trial Registration:**

ClinicalTrials.gov NCT04889794; https://clinicaltrials.gov/ct2/show/NCT04889794

**International Registered Report Identifier (IRRID):**

DERR1-10.2196/42577

## Introduction

The latest global figures show that 55 million persons lived with major neurocognitive disorders (MNCDs) in 2021, and this number is expected to rise to 78 million in 2030 and 139 million in 2050 [1]. Older adults with MNCDs are more likely to be exposed to polypharmacy or potentially inappropriate medications than those without this condition [2]. Polypharmacy, the simultaneous use of 5 or more different medications, increases older adults’ vulnerability to falls, emergency department visits, hospitalizations [3], and loss of autonomy [4]. For example, adverse effects of medications can be a precipitating factor in delirium [5]. In turn, delirium increases the risk of prolonged hospitalization and functional decline and doubles the mortality rate [6]. Better pharmacotherapy management for vulnerable older adults may prevent this iatrogenic chain leading to autonomy loss. According to several recent studies and literature reviews, interdisciplinary interventions that include pharmaceutical care and knowledge exchange sessions with health care teams can reduce inappropriate medication [7,8] or prevent the onset of delirium for at-risk individuals [9].

Since October 2015, the Quebec Ministry of Health and Social Services has provided resources to integrate pharmacists into interdisciplinary family medicine group (FMG) teams to improve medication use [10]. These pharmacists are working in the clinic, together with other health care professionals, in the provision of direct patient care. As of April 2021, 328 (88.6%) of the 370 FMGs had an agreement in place with 1 or more pharmacists, according to the Ministry. The pharmacist presence in the FMG was a mean of 16.3 and 19.1 hours per week in 2018 and 2020, respectively [11,12]. In January 2021, their role was further expanded by increased practice rights [13]. Notably, pharmacists can now initiate or modify a pharmacotherapy independently under certain conditions and, even more liberally, within a collaborative practice agreement with physicians [13].

Pharmacists are the health care professionals best trained to identify inappropriate medications [14], thus improving prescribing, reducing evitable health care utilization and medication costs, and contributing to the clinical improvement of many health conditions [14-16] and patients’ experience [17], specifically when part of the health care teams. Additionally, FMG pharmacists are particularly well positioned to review and optimize medications for patients undergoing cognitive assessment who frequently have inappropriate medication therapy [18]. A prior study examined this new model of functioning for pharmaceutical care in FMG among persons with complex care needs [19]. According to this study, the mean number of medications prescribed per person was 14.2, and FMG pharmacists identified an average of 7.2 problems related to pharmacotherapy during their evaluation and follow-up of 4 to 6 months [19]. A prior study in Quebec long-term care facilities on the increased role of pharmacists practicing in these settings demonstrated that it was possible to reorganize care, based on expanded pharmacists’ and nurses’ scope of practice, to reduce polypharmacy and inappropriate medications in older adults in long-term care [20]. Collaboration and work satisfaction among health care providers also improved [21,22].

Presently, not all FMGs have a pharmacist in their team, and when present, the FMG pharmacist is not always involved in the care trajectory of older adults with MNCDs. This study, therefore, aims to involve FMG pharmacists systematically in the care of these patients, a role which has been encouraged by the Quebec Alzheimer Plan [23,24]. This study will assess whether pharmacists’ systematic involvement in these patients’ care, together with their increased practice roles, can reduce treatment burden, polypharmacy, or potentially inappropriate medications and improve patients’ care, their satisfaction with care, and quality of life. We developed the intervention, called GPS (Évaluation de l’impact de la réorganisation du travail en GMF sur la pharmacothérapie et le soutien à l’autonomie des personnes âgées ayant un trouble neuro-cognitif majeur) together with the stakeholders of the Quebec Alzheimer Plan. Patient partners have also been involved in the study from its beginning and helped develop all recruitment and interviewing material. The intervention’s goal is to reduce the number of adverse effects due to inappropriate pharmacotherapy and its consequences on functional decline and loss of autonomy in older adults with MNCDs. We hypothesize that the intervention will decrease (1) the number of prescribed medications, (2) the proportion of patients with potentially inappropriate medication, and (3) patients’ perceived treatment burden and increase (4) patients’ satisfaction with care and (5) their quality of life.

## Methods

### Study Design

We based our methodology on the Medical Research Council's conceptual model on how to evaluate complex interventions [25]. We will use mixed methods, including a quasi-experimental controlled trial (step 1) and semistructured interviews (step 2). For step 1, information on health conditions and medication utilization at study entry and follow-up (ie, after 6 months) will be collected for all included participants. Participating patients will also complete 3 validated questionnaires in either French or English to assess their perceived treatment burden, quality of life, and satisfaction with care. The Multimorbidity Treatment Burden Questionnaire, (MTBQ) [26,27], the EQ-5D-5L [28], and the self-administered Physician Enabling Skill Questionnaire (PESQ) [29] will be used. Pharmacists will also report their interventions and suggestions during the 6-month follow-up. In step 2, we will invite all health professionals who implemented the intervention (ie, pharmacists, nurses, and physicians) to participate in a semistructured interview where they will be asked about the ease to integrate the intervention into their practice, their satisfaction with it, and their perception of its impacts for older adults and their families.

### The GPS intervention

First, nurses and physicians in intervention FMGs (exposed) will be asked to refer all older adults undergoing cognitive evaluation OR referred to a memory clinic OR recently diagnosed (<12 months) with a MNCD to the FMG pharmacist. Moreover, home care teams will be asked to refer older adults receiving home care for MNCDs to the FMG pharmacist. FMG pharmacists will perform a medication review. A medication review is a structured and comprehensive evaluation of a patient’s pharmacotherapy with the aim of identifying and resolving problems and improving health outcomes [30]. This medication review involves an interview with each referred older adult, including their caregiver, if applicable, to establish the best possible medication history (BPMH) [31]. The BPMH is a complete documentation of medication therapy, including the name, dose, administration route, and medication administration frequency. To this end, medication information will be validated with at least 1 other reliable data source (eg, community pharmacy records) and documented in the patient’s chart. The pharmacist will then detect medication-related problems [32] by analyzing this information and clinical data using their own judgment or validated criteria [33]. They will establish a care plan and the follow-ups needed with the patient in collaboration with the health care team and the community pharmacist. Pharmacists will also document the number and type of interventions and recommendations made to optimize patients’ pharmacotherapy for 6 months following the medication review. We will offer training and mentoring support to these teams, including pharmacist support for more complex interventions and monthly web-based interdisciplinary meetings.

### Study Setting

The study will comprise participants from an exposed group of approximately 5 FMGs with attending pharmacists who will implement the intervention and a control group composed of participants from 5 others FMGs without involved pharmacists. FMGs and home care teams will participate at their convenience within 2 Integrated (University) Health and Social Services Centers territories: (1) the *Centre intégré universitaire de santé et de services sociaux* (CIUSSS) *du Nord-de-l’Ile-de-Montréal* (NIM) in a metropolitan area and (2) the *Centre intégré de santé et de services sociaux* (CISSS) *de Chaudière-Appalaches* (CA) in a mixed—urban and rural—area. We used this selection process to facilitate implementation as the intervention teams need to be motivated toward its application and training and mentoring activities. To be eligible for participation, intervention FMGs must have at least 1 participating pharmacist, interested partner nurses, and physicians. Similarly, intervention home care teams must have access to a participating FMG pharmacist and a home care nurse. In contrast, in control FMGs and their home care teams, no FMG pharmacist will be systematically involved in the eligible patients’ trajectory. However, the FMGs must be willing to allow data collection and delay implementing similar interventions within their teams.

### Study Population

For the first step, the intervention targets 2 patient subgroups at turning points: (1) older adults undergoing cognitive evaluation or recently diagnosed with MNCDs in FMGs and (2) older adults with MNCDs followed at home by the home care teams.

For the second step, FMG professionals and home care teams implementing the intervention are also targeted.

#### Step 1: Quasi-experimental Controlled Trial

##### Inclusion and Exclusion Criteria for Older Adults

All older adults (aged ≥65 years) undergoing cognitive evaluation at the FMG OR referred to a memory clinic OR having been diagnosed with MNCDs at the FMG within the last year OR followed up at home for a MNCD AND referred to the pharmacist in the intervention FMGs will be invited. We will exclude older adults in palliative care or those who do not understand French or English (without a caregiver who could assist them).

##### Sampling Method, Recruitment, and Data Collection Procedure

We will conduct a quasi-experimental (ie, rather than a randomized) study given that our intervention will depend on whether an older adult’s FMG includes a participating pharmacist (intervention group) or not (control group).

##### Sample Size Calculation

According to prior estimates provided by the CI(U)SSS, about 50 older adults may be targeted for participation per FMG per year. Considering that the on-site presence of FMG pharmacists is on average 2 to 3 days a week [11,12], it should be feasible to include approximately 40 older adults per FMG. By setting our intercluster correlation coefficient at 0.005 (conservative), the number of clusters (FMGs) at 5 intervention and 5 control FMGs, and at 40 older adults per FMG, we expect to be able to detect a difference of 1 medication per older adults between the beginning and the end of the intervention, between the 2 groups, with a power of 83%. Therefore, we have planned a 12-month inclusion period, corresponding to recruiting approximately 1 person per week per FMG. The inclusion date will be when the reference to the pharmacist was made. For control FMGs, we will consult electronic medical records and FMG health care professionals to identify all older adults who meet the inclusion criteria. Since there will be no reference to the pharmacist for these individuals, the inclusion date will be the date of identification by the FMG. For those receiving home care services, the inclusion date will be the first home visit following the start of the study.

##### Outcomes Measures

The primary outcome measure is the change in the number of prescribed medications. Specifically, (1) the total number of prescribed medications and (2) the number of potentially inappropriate medications according to the Beers criteria [33] will be measured in the intervention and control groups. We will obtain data from patients’ medical records at the beginning (baseline) and 6 months after inclusion in the study.

We will assess several secondary outcome measures. First, a change in the treatment burden level will be measured with the 13-item MTBQ [26,27] in the intervention and control groups at baseline and 6 months follow-up. We will score each item as follows: 0 (not difficult/does not apply), 1 (a little difficult), 2 (quite difficult), 3 (very difficult), and 4 (extremely difficult). We will interpret the scores as suggested by the authors of the original MTBQ instrument: no burden (score 0), low burden (score <10), medium burden (score 10-22), and high burden (score ≥22) [26].

Second, we will measure the quality of life in the intervention and control groups at baseline and 6 months follow-up with the EQ-5D-5L [28]. The descriptive system comprises 5 dimensions: mobility, self-care, usual activities, pain/discomfort, and anxiety/depression. Each dimension has 5 levels: no problems, slight problems, moderate problems, severe problems, and extreme problems. Responses are coded as single-digit numbers expressing the severity level selected in each dimension [28]. The last study outcome is the satisfaction with the care received and access to care. To measure this outcome, the PESQ, validated in Quebec primary care settings, will be used [29]. We have adapted the questions with the author’s approval to consider not only the physician but the whole FMG team.

#### Step 2: Semistructured Interviews—Recruitment and Information-Gathering Procedures

The research team will invite all intervention FMG and home care professionals involved in the GPS intervention (pharmacists, nurses, and physicians) to an individual telephone interview at the end of the implementation period (ie, 18 months after inclusion of the first older adults). We will obtain verbal consent from the health care professionals before conducting the interview, which will last 30 to 45 minutes. We will aim for a total of approximately 30 interviews—that is, at least 1 pharmacist, 2 FMG nurses, and 2 physicians per FMG (n=25) and 6 nurses or nursing assistants from the home care team. This number should be sufficient to gather all the various health care professionals’ experiences while being feasible in the context of FMGs. A qualified interviewer will conduct the interviews using a semistructured interview guide to explore their views on the intervention. The interview will cover categories proposed by Patton’s Theoretical Model of Change [34]: (1) resources required for support, training, and coaching; (2) activities required by the model; (3) participation in the model’s implementation; (4) reactions to and satisfaction with the model; (5) changes in knowledge, attitudes, and skills; (6) change in practice; and (7) perceived results of the model. We will also invite professionals to complete the pretested French version of the web-based NoMAD instrument [35,36] derived from the Normalization Process Theory [37] after the end of the implementation period.

### Data Analysis

In step 1, we will use an intention-to-treat approach to assess the GPS intervention’s effectiveness. The principal analysis will compare the difference (1) in the average number of medications per person between the unexposed and exposed groups according to the study period (baseline and 6 months) and (2) the average number of potentially inappropriate medications per person between the 2 groups, according to the study period, by mixed Poisson repeated measures regression. This type of analysis considers intra-FMG and intraindividual correlations. Contrasts will be built to determine if the average number of medications per person over time differs between the exposed and the unexposed group. Secondary results will be analyzed similarly to compare the MTBQ, PESQ, and EQ-5D-5L scores between the groups exposed and unexposed to the GPS intervention.

Medication data missing at measurement time periods after the initial data collection will be imputed using the last-observation-carried-forward method. Using this method, the potential benefits of the intervention should be underestimated. For participants lost to follow-up regarding the questionnaires, no measures will be imputed at follow-up, but sensitivity analyses may be used in which baseline questionnaires scores could be imputed for follow-up scores. We will perform all analyses with SAS statistical software (SAS Institute Inc).

In step 2, we will record the interviews and transcribe them verbatim to then perform qualitative thematic content analysis. The first stage of this analysis consists of coding the data using NVivo qualitative analysis software (QSR International). We will develop a codebook based on Patton’s Theoretical Model of Change [34] for this purpose. Two research agents will independently test the first version of the codebook by coding excerpts of a few interviews. They will discuss and improve this version and test it again by coding excerpts of other interviews. This procedure aims to obtain a rigorous coding of the interviews and an accurate description of the experiences. The final sequential analysis will allow the integration of the quantitative results on the intervention’s impacts with the qualitative results describing the experiences of the FMG and home care teams. We will build and present an integration matrix and discuss it with the members of the research team and the stakeholders with whom the project was initiated, as well as with patient partners. This process will identify crucial elements for improving the intervention for future implementation.

According to mixed methods research methodology, the quantitative results obtained at step 1 will be interpreted and discussed in the light of information obtained from step 2, so that lessons can be learned on how to best implement such an interdisciplinary practice change, including the increased roles of some of the players [38].

### Ethics Approval

Ethical approval was obtained from the Ethics review board of the CISSS CA and CIUSSS NIM (project number: MP-23-2020-732; latest amendments approved in January 2022).

### Safety Consideration

The participant’s consent to participate in the study will be obtained in writing or verbally, recorded using an audio device. All participants will be able to withdraw from the study at any time without giving reasons. All patient data will be collected and recorded into REDCap, a secure and confidential data entry software. Nominal patient data will be entered into a data collection sheet and confidentially stored in an ongoing computer database of the research center (*CHU de Québec*).

## Results

All study materials (questionnaires, patients’ recruitment tools, and training documents) have been developed in collaboration with clinicians and patient partners. As of September 2022, 13 FMGs have agreed to participate in the GPS project (11 implementing the intervention and 2 as controls). We have yet to recruit more FMGs that will be part of the control group. Recruitment of older adults began in September 2021, when the project was launched. We had planned 12 months for the inclusion of eligible older adults. As of September 2022, a total of 100 participants had been enrolled and the follow-up was completed for 23 of them. Data collection will take approximately 18 months, and data analysis and synthesis of the results will take another 9 months. Knowledge transfer/mentoring sessions will be organized regularly during the implementation period (ten 1-hour meetings in each CI[U]SSS have been realized as of September 2022) and are also planned after the end of the GPS study (approximatively 6 months). These sessions are open to FMG pharmacists, physicians, mentors (physicians and pharmacists with geriatric expertise), and research team members and comprise short presentations of specific clinical interest, summaries of issues surrounding the study methods and procedures, as well as an occasion for an exchange between all these persons on clinical and research questions.

## Discussion

Our hypothesis is that the GPS intervention will improve pharmaceutical care for older adults undergoing cognitive assessment or with MNCDs and facilitate access to care. This research will add new knowledge on the impact of a systematic involvement of pharmacists in FMGs and their home care teams for older adults with MNCDs. In fact, despite growing recognition of the urgent need to address the “epidemic of polypharmacy in geriatric patients,” this study is one of the first to evaluate the impact of an interdisciplinary care model involving FMG pharmacists on the pharmacotherapy of older adults with MNCDs. It will also evaluate the processes required to implement the GPS intervention and develop tools, procedures, and guidelines that could be transposed to other care settings to improve care and its continuity for these patients.

This study received financing as a “living laboratory,” meaning that the proposed intervention will be adapted by the participating FMGs or home care teams and may thus vary slightly according to the different contexts of practice environments. This variability could influence the internal validity of the results, which is a possibility common in such strategies. Additionally, the COVID-19 pandemic has caused delays in deploying the project across the FMGs and brought some difficulties in patient recruitment. It might also be more challenging to recruit further FMGs because health care professionals must be willing to invest some time in implementing the intervention or for recruiting participants. Moreover, the characteristics of participants at risk or with a MNCD (eg, fatigue, memory loss, attention difficulties, anxiety, etc) may affect their level of understanding of their involvement in the project and possibly complicate data collection. Adjustments will be made in the methodology to account for the characteristics of the study population if necessary.

## Abbreviations

BPMH: best possible medication history
CA: Chaudière-Appalaches
CISSS: Centre intégré de santé et de services sociaux
CIUSSS: Centre intégré universitaire de santé et de services sociaux
FMG: family medicine group
MNCD: major neurocognitive disorder
MTBQ: Multimorbidity Treatment Burden Questionnaire
NIM: Nord-de-l’Ile-de-Montréal
PESQ: Physician Enabling Skill Questionnaire

## Data Availability

The data sets generated and analyzed during the current study are not publicly available due to data access and research ethics laws applicable in Quebec but are available from the corresponding author on reasonable request.
